# Conformational Occlusion of Blockade Antibody Epitopes, a Novel Mechanism of GII.4 Human Norovirus Immune Evasion

**DOI:** 10.1128/mSphere.00518-17

**Published:** 2018-02-07

**Authors:** Lisa C. Lindesmith, Michael L. Mallory, Kari Debbink, Eric F. Donaldson, Paul D. Brewer-Jensen, Excel W. Swann, Timothy P. Sheahan, Rachel L. Graham, Martina Beltramello, Davide Corti, Antonio Lanzavecchia, Ralph S. Baric

**Affiliations:** a Department of Epidemiology, University of North Carolina, Chapel Hill, North Carolina, USA; b Department of Natural Sciences, Bowie State University, Bowie, Maryland, USA; c Humabs BioMed SA, Bellinzona, Switzerland; d Institute for Research in Biomedicine, Bellinzona, Switzerland; e Institute of Microbiology, Zurich, Switzerland; Icahn School of Medicine at Mount Sinai

**Keywords:** adaptive immunity, blockade antibody, broadly neutralizing antibody, epitope shielding, evolution, immune evasion, monoclonal antibody, norovirus, particle dynamics

## Abstract

Extensive antigenic diversity within the GII.4 genotype of human norovirus is a major driver of pandemic emergence and a significant obstacle to development of cross-protective immunity after natural infection and vaccination. However, human and mouse monoclonal antibody studies indicate that, although rare, antibodies to conserved GII.4 blockade epitopes are generated. The mechanisms by which these epitopes evade immune surveillance are uncertain. Here, we developed a new approach for identifying conserved GII.4 norovirus epitopes. Utilizing a unique set of virus-like particles (VLPs) representing the *in vivo*-evolved sequence diversity within an immunocompromised person, we identify key residues within epitope F, a conserved GII.4 blockade antibody epitope. The residues critical for antibody binding are proximal to evolving blockade epitope E. Like epitope F, antibody blockade of epitope E was temperature sensitive, indicating that particle conformation regulates antibody access not only to the conserved GII.4 blockade epitope F but also to the evolving epitope E. These data highlight novel GII.4 mechanisms to protect blockade antibody epitopes, map essential residues of a GII.4 conserved epitope, and expand our understanding of how viral particle dynamics may drive antigenicity and antibody-mediated protection by effectively shielding blockade epitopes. Our data support the notion that GII.4 particle breathing may well represent a major mechanism of humoral immune evasion supporting cyclic pandemic virus persistence and spread in human populations.

**IMPORTANCE** In this study, we use norovirus virus-like particles to identify key residues of a conserved GII.4 blockade antibody epitope. Further, we identify an additional GII.4 blockade antibody epitope to be occluded, with antibody access governed by temperature and particle dynamics. These findings provide additional support for particle conformation-based presentation of binding residues mediated by a particle “breathing core.” Together, these data suggest that limiting antibody access to blockade antibody epitopes may be a frequent mechanism of immune evasion for GII.4 human noroviruses. Mapping blockade antibody epitopes, the interaction between adjacent epitopes on the particle, and the breathing core that mediates antibody access to epitopes provides greater mechanistic understanding of epitope camouflage strategies utilized by human viral pathogens to evade immunity.

## Abstract (precis)

In this study, we use norovirus virus-like particles to identify key residues of a conserved GII.4 blockade antibody epitope. Further, we identify an additional GII.4 blockade antibody epitope to be occluded, with antibody access governed by temperature and particle dynamics. These findings provide additional support for particle conformation-based presentation of binding residues mediated by a particle “breathing core.” Together, these data suggest that limiting antibody access to blockade antibody epitopes may be a frequent mechanism of immune evasion for GII.4 human noroviruses. Mapping blockade antibody epitopes, the interaction between adjacent epitopes on the particle, and the breathing core that mediates antibody access to epitopes provides greater mechanistic understanding of epitope camouflage strategies utilized by human viral pathogens to evade immunity.

## INTRODUCTION

Antigenic diversity is a hallmark of many successful RNA viruses. However, requirements for structure integrity and maintenance of key capsid functions such as receptor binding and cell fusion require essential residues to remain conserved over time, representing potential cross-protective antibody (Ab) epitopes (1–3). To combat this weakness, many human RNA viruses, including seasonal influenza virus (4), human immunodeficiency virus (5), hepatitis C virus (6), Ebola virus (7), West Nile virus (8), and human norovirus (9), have evolved strategies to camouflage neutralizing antigenic sites. Local mechanisms of camouflage include shielding of the epitope with carbohydrates (4, 10) or lipids (11, 12) or by structurally occluding the site by burying the epitopes beneath the surface topology (6, 13–15). Other mechanisms include “particle breathing,” described as dynamic conformational changes in the virion that limit antibody access to occluded epitopes (5, 7–9, 16, 17). Effective use of these evasive mechanisms provides an advantage to viruses with high population exposure, including human norovirus.

Human norovirus is the leading cause of acute gastroenteritis, causing more than 21 million infections per year in the United States and approximately 200,000 deaths worldwide, primarily in the young and aged populations (18). This heavy disease burden on particularly vulnerable populations warrants development of a norovirus vaccine. The primary obstacle to a successful vaccine is the extensive antigenic diversity between norovirus strains and within the pandemic GII.4 strains (19–21). Like influenza A virus, the major capsid sequence of the norovirus GII.4 strains is undergoing epochal evolution, resulting in emergent immune escape variants every 2 to 5 years (20, 22, 23). Using an *in vitro* surrogate neutralization assay to measure antibody blockade of norovirus virus-like particle (VLP) binding to carbohydrate ligand, shown to correlate with protection from infection, four evolving “blockade” antibody epitopes have been characterized (24, 25). Epitope A is immunodominant (~40% of the serum blockade antibody response) and changes with each epidemiologically significant strain (26–28). Epitope D lies along the ridge of the carbohydrate-binding domain and is both a blockade antibody epitope and a mediator of carbohydrate binding affinities (25). Epitopes A and D face the most exterior part of the viral particle (the P2 subdomain) and are easily accessible to potent blockade antibodies (9). Epitope E is lateral to epitope D and is less exposed to the surface (25, 29). Finally, epitope F is highly conserved across GII.4 strains, and its structural location is unknown (9, 25). Norovirus infection and vaccination elicit antibodies to subdominant epitope F. Antibody binding to epitope F is mediated by residues outside the antibody-binding site. Residues 310, 316, 484, and 493, the NERK motif, are highly conserved across GII.4 strains (9) and are located distal to the top surface of the particle where epitopes A and D are located. Incubation temperature and mutations in the NERK motif affect antibody access to epitope F by allosteric effects on particle conformation with an unclear mechanism (25, 30).

The goal of this study is to identify the GII.4 conserved blockade epitope recognized by human monoclonal antibody (MAb) GII.4F. The high degree of conservation of epitope F has limited the effectiveness of bioinformatic approaches to identifying epitope F and additional NERK motif residues, although this approach was instrumental to predicting evolving blockade antibody epitopes that were further verified by testing chimeric VLPs and MAbs (25, 26). Here, we used a unique set of reagents based on viral sequences isolated from an immunocompromised person with a long-term norovirus infection (31, 32) to identify key residues of a conserved GII.4 blockade antibody epitope. These residues were invariant in all other panels of GII.4 VLPs that we have studied thus far. In addition, we apply quantitative biochemical analyses to differentiate between residues that affect antibody binding (epitope) and residues that affect antibody access to the epitope through allosteric mechanisms (particle dynamics regulating domain). These findings provide new support for particle conformation-based presentation of key binding residues that are regulated by a “breathing core” which includes the NERK motif and an additional amino acid. Further, like epitope F, epitope E is demonstrated to be occluded, with Ab access governed by temperature and particle dynamics. These data indicate that limiting antibody access to blockade antibody epitopes may be a frequent mechanism of immune evasion for GII.4 human noroviruses. Mapping a blockade antibody epitope, the interaction between adjacent epitopes on the particle, and the breathing core that mediates antibody access to epitopes provides greater mechanistic understanding of epitope camouflage strategies utilized by human viral pathogens.

## RESULTS

### Residues 327 and 404 are key binding sites of the GII.4 conserved blockade antibody epitope.

**FIG 1:**
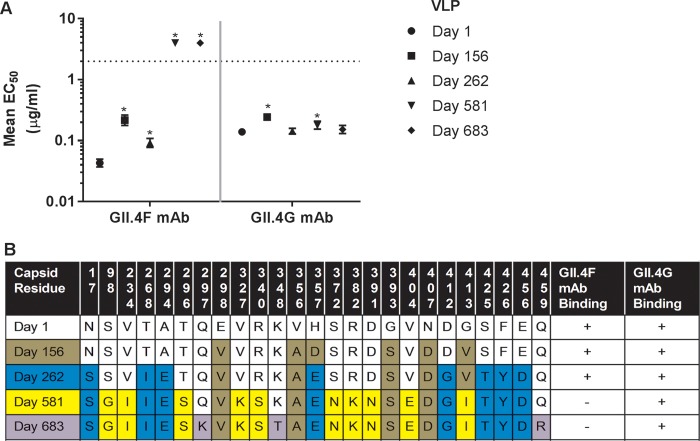
Long-term *in vivo* evolution results in loss of GII.4 epitope F, a conserved GII.4 blockade antibody epitope. (A) Virus-like particles representing norovirus sequences isolated from an immunocompromised transplant patient over a 683-day period were evaluated for binding to MAbs GII.4F and GII.4G by enzyme immunoassay. Both MAbs recognize conserved GII.4 blockade epitopes that overlap each other and are occluded by structural conformation. Error bars represent 95% confidence intervals. Values that are significantly different (*P* < 0.05) from the day 1 value are indicated by an asterisk. (B) After a minimum of 581 days of *in vivo* evolution, capsid sequence changes, indicated by color changes, resulted in loss of binding of human MAb GII.4F but not mouse MAb GII.4G.

**FIG 2:**
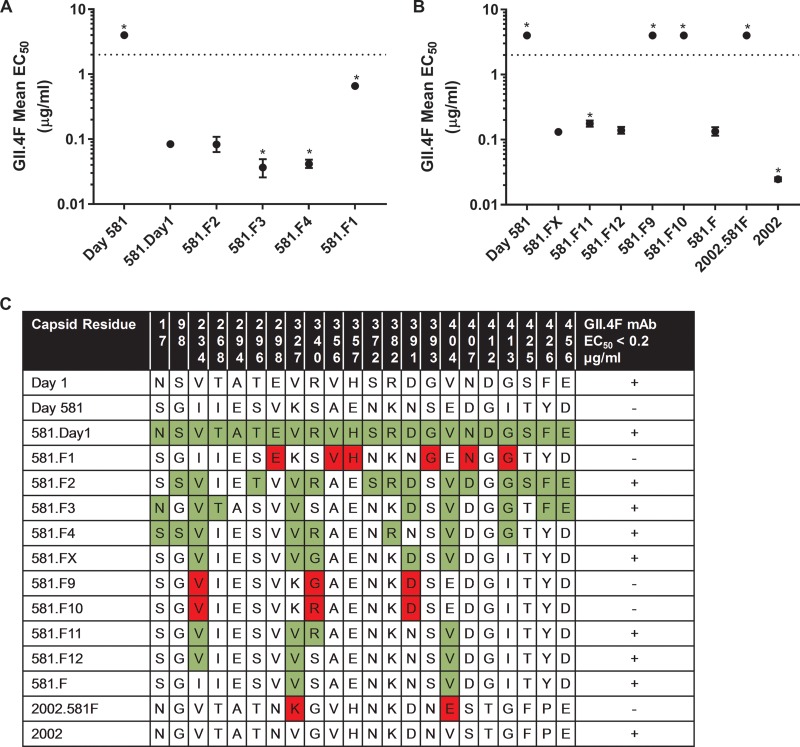
Residues 327 and 404 are key binding sites for GII.4F MAb. (A and B) To map the epitope for the GII.4F MAb, amino acid changes in the capsid protein between day 1 and day 581 strains were compared and sets of changes were introduced into the 581 backbone sequence. Chimeric VLPs were evaluated for MAb binding by EIA, and EC_50_ titers were determined. Values significantly different (*P* < 0.05) from 581.Day1 (A) or 581.FX (B) are noted with an asterisk. (C) Residues that were changed are shown in color to indicate gain (green) or loss (red) of GII.4F MAb binding. Changing valine at residues 327 and 404 resulted in an increase in binding of GII.4F MAb to 581 (581.F), while replacing valine at residues 327 and 404 in GII.4.2002 (2002.581F) resulted in a loss of binding of the GII.4F MAb.

**FIG 3:**
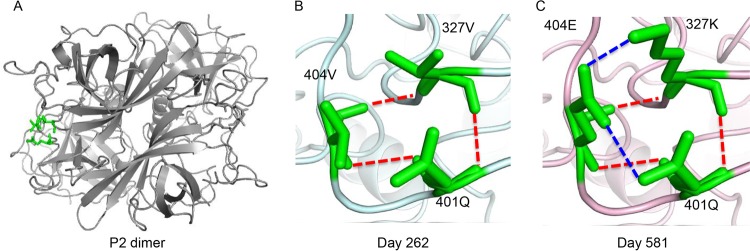
Homology models of epitope F interactions between days 262 and 581 viruses. (A) Amino acid positions 327, 401, and 404 are shown in green on the P2 dimer for context. (B and C) Three hydrogen bonds (red dashed lines) are evident among residues 327V, 401Q, and 404V in the day 262 model (B), while the original three hydrogen bonds (red dashed lines) plus two additional hydrogen bonds (blue dashed lines) are formed in the day 581 model (C) among 327K, 401Q, and 404E.

GII.4F or GII.4G MAbs recognize two spatially close conserved blockade epitopes with restricted access based on particle conformation (9). The spatial locations of targeted residues (Table 1) remain unknown, although NERK motif modifications in part regulate access to these epitopes (9). To map GII.4F or GII.4G residues, VLPs representing time-ordered GII.4.2006a norovirus strains that evolved within an immunocompromised transplant patient over 683 days (31, 32) were synthesized, expressed, and characterized for binding of GII.4F or GII.4G MAbs by enzyme immunoassay (EIA) (Fig. 1). GII.4G MAb appears to bind to a unique conserved epitope, designated epitope G, that may overlap epitope F and was preserved during the period of monitoring, as assessed by both binding and blockade assays. In contrast, by day 581, evolution in epitope F resulted in loss of binding of MAb GII.4F (Fig. 1A). Day 581 VLP is the first identified GII.4 VLP not to react with MAb GII.4F. Amino acid substitutions associated with *in vivo* evolution occurred at 22 residues within the major capsid protein between day 1 and 581 of monitoring (31). Fifteen of the changed residues were in the P2 domain, potentially influencing blockade antibody epitopes (Fig. 1B). To map the epitope for MAb GII.4F, amino acid changes in the capsid protein were compared, and sets of changes were introduced into the day 581 backbone sequence (Fig. 2). Restoring the 14 residues in 581.F2 resulted in an increase in the binding of GII.4F similar to the increase from restoring all 22 residues that differed between day 1 and day 581 (581.Day1) (Fig. 2A). 581.F3 and F4, but not 581.F1, improved GII.4F binding in EIA. A five-residue exchange of F2 residues (residues 234, 327, 340, 391, and 404) into the day 581 backbone (581.FX) resulted in an increase in GII.4F binding and was further analyzed (Fig. 2B and C). Only VLPs with valines at positions 327 and 404 were sufficient to restore GII.4F binding (581.F). Conversely, when changing V327 to K and V404 to E in GII.4.2002 (2002.581F), GII.4F binding was lost, confirming that V327 and V404 are critical key residues of the conserved GII.4 blockade Ab epitope F. Homology modeling of residues 327 and 404 shows that they form a conformational epitope proximal to evolving blockade antibody epitope E. In the model, K327 directly interacts with E404 via electrostatic interaction, which likely rearranges the local structural neighborhood of epitope F. In addition, modeling of the hydrogen bond networks between residues V327, Q401, and V404 suggests that three hydrogen bonds are formed in the day 262 isolate (Fig. 3, red dashed lines), while two additional hydrogen bonds (blue dashed lines) are formed in the day 581 model among 327K, 401Q, and 404E. These additional bonds likely contribute to the loss of GII.4F binding to day 581 VLPs.

**TABLE 1:** Characteristics of monoclonal antibodies used in this study

MAb	Synonym(s)	Species	Immunogen	Epitope
GII.4E	GII.4.2002.G6	Mouse	GII.4.2002	Variable, blockade epitope E of GII.4.2002
GII.4F	NVB 71.4	Human	Natural infection	Conserved GII.4, blockade, conformation dependent, epitope F
GII.4G	GII.4.2002.G5, MAB227P	Mouse	GII.4.2002	Conserved GII.4, blockade, conformation dependent, epitope G

### Residue 234 influences GII.4F MAb access to the conserved blockade antibody epitope.

**FIG 4:**
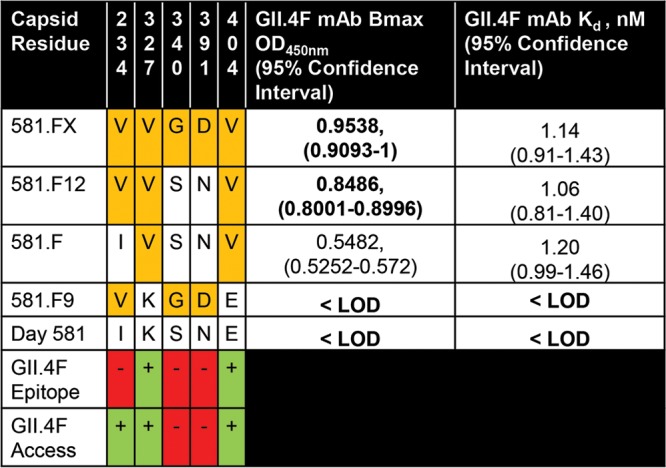
GII.4F antibody access to epitope F is influenced by residue 234, a residue outside the epitope. GII.4F MAb EC_50_ titer is mediated by both the maximum number of binding sites available (*B*_max_) and the relative affinity of the antibody for the epitope. To distinguish the effects of the five residues comprising 581.FX, chimeric VLPs containing admixtures of residues (orange) were tested for GII.4F MAb binding by EIA, and *B*_max _and *K*_*d*_ values were calculated. Changes in residues 340 and 391 did not change GII.4F MAb binding or access (red). The *K*_*d*_ for the epitope was consistent between the VLPs with valine at positions 327 and 404, identifying these residues as part of the GII.4 epitope F (green). In comparison, 234V was not associated with changes in *K*_*d*_ (red). The 234V VLPs had higher *B*_max_ values than the 581.F VLPs, indicating that residue 234 may be influencing GII.4F MAb binding via long-range allosteric effects, as described for the NERK motif. Bold values are significantly different (*P* < 0.05) from the values for 581.F. <LOD, less than the limit of detection.

**FIG 5:**
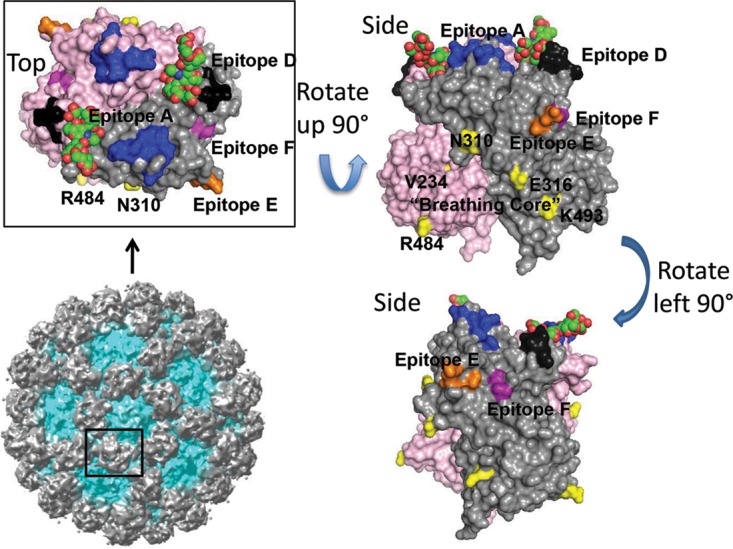
Residues important for mediating GII.4 norovirus antigenicity. A homology model of a P2 domain dimer (light gray and magenta monomers) of GII.4.2006a bound to A antigen (red and green) with identified blockade antibody epitopes A (blue), D (black), E (orange), and F (purple) and the “breathing core” residues (NERK plus residue 234) that mediate global particle conformation (yellow) color coded. The full GII.10 VLP, in which P dimers are shown in gray, is shown for context.

As previously described, GII.4F MAb blockade potency is mediated by both the number of accessible binding sites as well as by the affinity of the antibody for the target epitope (9). To understand the contributions to binding of the five residues included in 581.FX, chimeric VLPs containing admixtures of residues 234, 327, 340, 391, and 404 (581.F9-F12) were tested for GII.4F MAb binding by EIA (9, 33). VLPs are composed of 90 copies of a dimer of the capsid protein, accounting for a total of 180 copies of nonquaternary epitopes. Changes in the maximum number of binding sites (*B*_max_) reflect access to binding of antibody to the these epitopes (34). *K*_*d*_ (dissociation constant, relative affinity) is dependent on the strength of molecular interaction between the epitope and the antibody. Changes in residues 340 and 391 did not affect GII.4F MAb relative affinity or access (Fig. 4). The *K*_*d*_ was consistent between the VLPs with valines at positions 327 and 404 (1.1 nM), indicating that these residues define part of GII.4 epitope F. V234 was not associated with changes in *K*_*d*_. The V234 VLPs (581.FX and 581.F12) (Fig. 4) had higher *B*_max_s than 581.F (K327V and E404V), indicating that residue 234 may be influencing GII.4F MAb binding via long-range allosteric effects, as described for the NERK motif (9). The NERK motif and residue 234 (the “breathing core”) are distal to the epitope and near the dimer interface (Fig. 5).

### Antibody access to evolving blockade antibody epitope E depends on global particle and local conformation.

**FIG 6:**
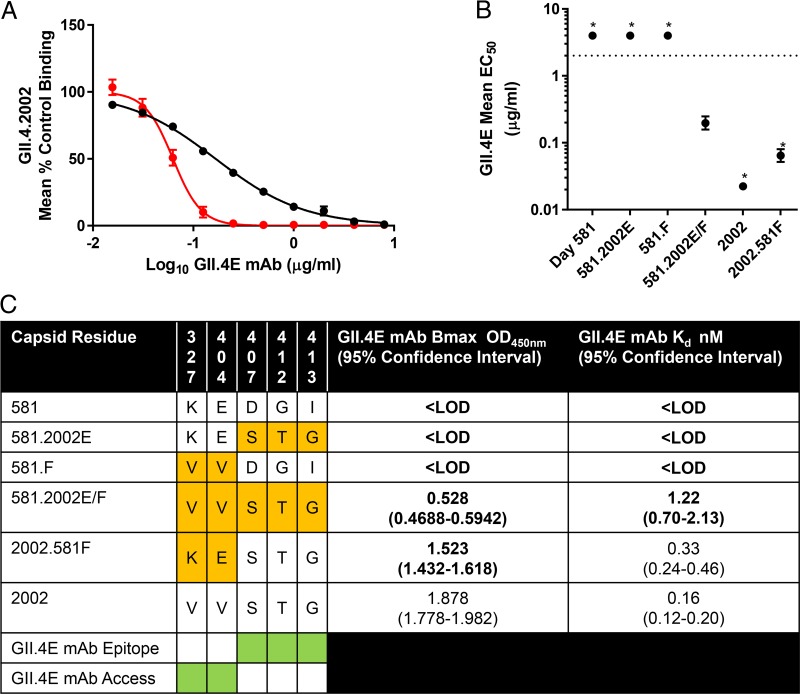
GII.4E MAb binding is regulated by global particle conformation and surrounding local topology. (A) GII.4.2002 VLP binding to ligand was blocked by GII.4E at either room temperature (black) or 37°C (red), and mean percent control binding compared to no antibody was fit by sigmoidal dose-response curve analysis, with the Hill slopes and EC_50_s calculated. Incubation at 37°C resulted in a steeper curve and lower EC_50_ titer, indicating that antibody access to epitope E is dependent upon particle conformation, as described for epitopes F and G. (B) Exchange of both epitope E and F residues into the 581 backbone (581.2002E/F) improved binding of GII.4E MAb compared to exchanging only either epitope E (581.2002E) or epitope F (581.F) residues. Values that are significantly different (*P* < 0.05) from the value for 581.2002E/F are indicated by an asterisk. (C) To distinguish the effects of epitope E and F residues on GII.4E binding, chimeric VLPs (orange) were tested for GII.4E binding by EIA, and *B*_max_ and *K*_*d*_ values were calculated. Exchange of day 581 epitope F residues into the 2002 backbone (2002.581F) did not change the *K*_*d*_ for GII.4E but did decrease the *B*_max_, indicating that epitope F residues mediate GII.4E epitope access. In support of this hypothesis, exchange of both epitope E and F residues from the 2002 backbone into the 581 backbone (581.2002E/F) was necessary to restore GII.4E binding. Bold values are significantly different (*P* < 0.05) from GII.4.2002. Green shading indicates residues involved in GII.4E epitope binding and access to the epitope. LOD, less than the limit of detection.

Epitope E, like epitope F, is lateral to epitopes A and D, which line the outermost surface of the viral particle (25). Epitope E and F residues are within 7 to 23 Å of each other. To determine whether particle conformation also regulates antibody access to epitope E, GII.4.2002 blockade by MAb GII.4E (Table 1) (29) was tested at room temperature and 37°C, as previously described (Fig. 6) (9). Increased temperature of incubation resulted in a 2.7-fold decrease in 50% effective concentration (EC_50_) titer and a 3.2-fold increase in blockade curve slope for GII.4E blockade of GII.4.2002 (Fig. 6A), indicating that GII.4E access to evolving blockade epitope E improves at higher temperature. These results are similar to those reported for epitope F and G blockade and suggest that particle dynamics also regulate antibody access to epitope E (9).

To explore any direct or indirect interaction between epitopes E and F, a series of VLPs with exchanged epitope E, epitope F, or both were tested for EIA binding with MAb GII.4E (Fig. 6B). Exchange of either epitope E (581.2002E) or F (581F) residues from GII.4.2002 into the 581 backbone did not confer GII.4E binding. However, exchange of both epitopes E and F (581.2002E/F) resulted in an increase in GII.4E binding. Conversely, exchange of epitope F residues from 581 into the 2002 (2002.581F) backbone increased 2.9-fold the EC_50_ titer of GII.4E for GII.4.2002. These data indicate that epitope F residues affect the binding of epitope E antibodies. To distinguish the effects of epitope E and F residues on GII.4E binding, chimeric VLPs were tested for GII.4E binding by EIA, and *B*_max_ and *K*_*d*_ values were calculated (Fig. 6C). Exchange of day 581 epitope F residues into the 2002 backbone (2002.581F) did not change the *K*_*d*_ (0.33 nM) for GII.4E but did decrease the *B*_max_. Therefore, epitope F residues are not part of epitope E but instead regulate GII.4E epitope access. These data indicate that local particle conformation also regulates antibody access to blockade epitopes.

## DISCUSSION

Blockade antibody responses are proposed correlates of human norovirus protective immunity (35). Elucidation of the specific epitopes correlated with protection could facilitate vaccine design strategies (36, 37). Human norovirus infection elicits a skewed blockade antibody response to the hypervariable epitope A of the infecting strain (9, 26). Similarly, in human trials, norovirus multivalent VLP vaccination recalls a memory response to previous strain epitope A (30). The ability to harness preferential antigen presentation on VLP vaccines could selectively drive vaccine immune responses away from the hypervariable epitopes and toward cross-protective conserved epitopes (10, 38). This report is the first to map key amino acids comprising part of a conserved GII.4 norovirus blockade antibody epitope (epitope F) recognized by a human monoclonal antibody, identify two additional conformation-dependent epitopes (epitopes E and G), and expand the set of residues that influence particle dynamics and antibody access to occluded blockade antibody epitopes. These are important steps in designing an engineered cross-protective norovirus VLP vaccine candidate.

The implications of norovirus infection in immunocompromised individuals is an expanding area of study. Virus sequencing from serial stool samples indicates that even under the atypical (reduced) immune pressure exerted in an immunocompromised patient, norovirus continues to evolve via antigenic drift at blockade antibody epitopes (Fig. 1) (32). This process mimics viral evolution within the general population that leads to the emergence of new viruses (20, 23). An extended period of *in vivo* evolution allowed the first identified change in epitope F. Valine at positions 327 and 404 are part of epitope F and are conserved across GII.4 norovirus strains circulating from 1974 to 2015. Occluded epitopes of other human RNA viruses are essential for receptor binding, viral fusion, and capsid assembly/disassembly, functions essential for infection/replication (1–3). It is unknown whether particle conformation plays similar roles in the norovirus life cycle or whether antibody binding to epitope F affects particle dynamics necessary for ligand binding or sterically blocks ligand interaction. The primary limitation to this study is the lack of a reverse genetics system to test these possibilities. The newly developed human norovirus replication system (39) may someday be amenable to a reverse genetics approach to studying viral mutants and elucidate the roles of epitope F residues. It is possible that V327 and V404 are not part of epitope F, but instead, mutations at these residues such as those observed in this study could prevent conformational transitions necessary for antibody binding. However, this explanation is unlikely, as antibody access to the overlapping epitope G and the neighboring epitope E remains temperature sensitive, indicating that particle plasticity is maintained in the 581 VLP (327K.404E).

Unlike residues 327 and 404, residue 234 is buried near the dimer interface and does not participate in GII.4F binding. Instead, residue 234 regulates antibody access to occluded epitopes by mediating particle conformation, similarly to what has been described for residues 310, 316, 484, and 493 (NERK motif) (9). These data indicate that residue 234, the NERK motif, and possibly other unidentified amino acids form a “breathing core” that works in concert to regulate global particle structure driving antigenicity and ligand binding (Fig. 5). Similarly, recent detailed bioinformatic studies with a monoclonal antibody with broad mouse norovirus neutralization potency identified a single amino acid outside the antibody-binding site that mediated antibody binding by changing particle conformation (40). A mutation distal to the surface altered interfacial interactions between the P domains of the dimers affecting the structure throughout the P domain. These data support previous findings on the NERK motif on human norovirus particle dynamics and the allosteric effect distant residues can have on antibody binding and suggest conformation-based epitope camouflage may be a mechanism of immune evasion conserved across noroviruses. Mutations in the breathing core could modulate epitope presentation, altering the effectiveness of antibody responses in protection from infection and influencing the repertoire of antibodies made following vaccination and infection (38). The NERK motif is highly conserved in GII.4 strains from 1974 to 2006. Contemporary strains GII.4.2009 and 2012 both introduced mutation at residue 310, which resulted in altered antibody access to epitope F (9). Although speculative, changes at residue 310 may have been driven by pressure to alter protection of epitope F. Further, controlling particle dynamics may have practical implications for VLP immunogens where viral entry is not maintained. An engineered VLP designed to have decreased fluctuation by temperature could improve thermostability and shelf life of VLP-based vaccines (41), as well as enhance antigen presentation stability (42).

On the basis of our existing panels of antibodies, presentation of epitopes A and D is not dependent on the particle conformation and changes in the NERK motif do not affect blockade potency of antibodies to these epitopes (9). The findings presented here indicate that the delineating factor between occluded and nonoccluded epitopes is likely location on the viral particle. Evolving blockade epitope E lies near the transition from the most-surface-exposed P2 subdomain of the capsid protein and the less-surface-exposed P1 subdomain. Epitope E is variable between pandemic strains, and antibody access is occluded (25, 29). Although a virion has the same number of epitopes A, E, F, and G, antibodies to epitopes E, F, and G are rare, compared to epitope A. Only a single monoclonal antibody to each epitope has been characterized thus far (25, 29), possibly reflecting immune suppression mechanisms that influence the antigenicity of these epitopes. The coordinates of epitope G are unknown, but like epitope E, are nearby and/or overlapping with epitope F (9). Mutation in the breathing core and temperature sensitivity of antibody blockade indicate that antibody access to epitopes E, F, and G is mediated at the global particle level. The observation that exchange of both epitope E and F residues from the 2002 backbone into the 581 backbone (581.2002E/F), but not epitope E residues alone, was necessary to increase GII.4E binding, indicates that antibody access is also mediated at the local level by the conformation of surrounding residues (43). These conformation-dependent regulatory mechanisms are likely to extend to other epitopes within the P1 subdomain. Additional human monoclonal antibodies resulting from norovirus infection and vaccination are needed to identify other epitope signatures and the interdependence of distinct epitopes within a continuous antigen.

**FIG 7:**
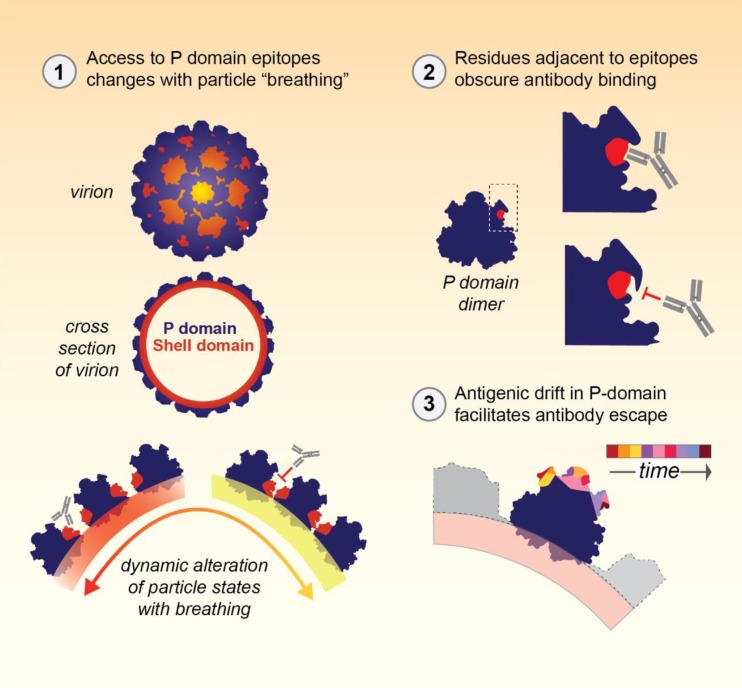
Sequence and spatial flexibility within the capsid of GII.4 noroviruses mitigates antibody-mediated protective immunity. Three mechanisms of immune evasion are proposed here. First, antibody binding to epitopes can be occluded by steric hindrance resulting from particle contraction during “breathing” (epitopes E, F, and G). Second, antibody binding to epitopes can be occluded by steric hindrance resulting from local particle conformation (epitopes E and F). Third, antigenic drift in highly antigenic, surface-exposed epitopes (epitopes A, D, and E) can facilitate antibody escape over time.

These studies expand our understanding of the complex mechanisms of human norovirus immune evasion (Fig. 7) and persistence in human populations. In addition to immune-driven selective pressure on a subset of residues to change (antigenic drift), global particle structure (mediated by the distant breathing core residues) and local particle structure (mediated by closely surrounding residues) impact antibody access to blockade epitopes. These topological changes effectively reduce antibody binding without requiring changes in the residues that comprise the actual epitopes. These structure conformation-based immune evasion strategies are particularly advantageous for protecting essential conserved motifs that could be targeted by antibodies. Norovirus joins other successful human pathogens such as HIV (5), influenza virus (10), Ebola virus (7), West Nile virus (8), and poliovirus (14) that use conformation-based shielding of key essential residues to evade development of protective immunity. Further study is needed to test the effects of breathing core mutations on presentation of epitopes E, F, and G. Concurrent with modifying the breathing core to change epitope access, surface residues that sterically block antibody access to the occluded epitopes may be identified. Changing these surface residues may be an easier path to design a VLP immunogen with preferential presentation of conserved epitopes and better cross-reactivity with emergent GII.4 strains (6, 36), a primary goal for norovirus vaccinology. These concepts that characterize how viral particle dynamics influence antigen presentation and antibody access to blockade epitopes may be applicable to vaccine strategies for other highly penetrant, antigenically diverse viruses.

## MATERIALS AND METHODS

### Virus-like particles.

Synthetically derived (Bio Basic Inc., Amherst, NY) ORF2 genes were inserted directly into the Venezuelan equine encephalitis replicon vector. Virus-like particles (VLPs) were expressed in baby hamster kidney cells (ATCC CCL-10TM) and purified by velocity sedimentation in sucrose (44). VLP protein concentrations were determined by the BCA protein assay (Pierce, Rockford, IL). Uranyl acetate-stained VLPs were visualized by transmission electron microscopy (see Fig. S1 in the supplemental material).

10.1128/mSphere.00518-17.1 FIG S1  Electron micrographs of epitope-mutant VLPs designed for this study. Download FIG S1, TIF file, 2.7 MB. Copyright © 2018 Lindesmith et al. 2018 Lindesmith et al. This content is distributed under the terms of the Creative Commons Attribution 4.0 International license.

### Enzyme immunoassay.

The wells on enzyme immunoassay (EIA) plates were coated with 0.25 μg/ml VLP in phosphate-buffered saline (PBS) for 4 h and blocked overnight at 4°C in 5% dry milk in PBS containing 0.05% Tween 20 (PBS–0.05% Tween 20) before the addition of decreasing twofold serial dilutions of monoclonal antibody (MAb). Bound MAb was detected by anti-human or mouse IgG conjugated to horseradish peroxidase (anti-human/mouse IgG-HRP) (GE Healthcare) and color developed with 1-Step Ultra TMB ELISA HRP substrate solution (Thermo Fisher). Each step was followed by washing with PBS–0.05% Tween 20, and all reagents were diluted in 5% dry milk in PBS–0.05% Tween 20. All incubations were done at 37°C. To determine 50% effective concentrations (EC_50_s) for antibodies with optical densities (ODs) ≥ 3× background at 2 µg/ml, EIA data were log transformed and fit using sigmoidal dose-response analysis of nonlinear data in GraphPad Prism 7.02 (GraphPad Software, La Jolla, CA) (9). Monoclonal Abs below the limit of detection were assigned an EC_50_ of 2× the assay upper limit of detection for statistical comparison. EC_50_s between VLPs were compared using the one-way analysis of variance (ANOVA) with Dunnett’s posttest. A difference was considered significant if the *P* value was <0.05. *B*_max_ and *K*_*d*_ values were estimated by one-site specific binding nonlinear curve fit of mean OD values in GraphPad Prism 7.02 (9, 33).

### Antibody blockade of VLP binding assay (blockade).

VLPs (0.25 μg/ml) were pretreated with decreasing concentrations of MAb for 1 h and added to wells coated with pig gastric mucin type III (Sigma-Aldrich, St. Louis, MO) for 1 h. Bound VLP was detected as described above using anti-VLP rabbit hyperimmune sera. Percent control binding is defined as the level of binding in the presence of antibody pretreatment compared to the level of binding in the absence of antibody multiplied by 100. The blockade data were fit using sigmoidal dose-response analysis of nonlinear data in GraphPad Prism 702. EC_50_ and Hill slope values were calculated for antibodies that demonstrated blockade of at least 50% at the dilution series tested. Antibodies that did not block 50% of binding at the highest dilution tested were assigned an EC_50_ of two times the assay upper limit of detection for statistical comparison (9). Increasing antibody-VLP-ligand incubations to 40°C (simulating fever) decreases the blockade EC_50_ about twofold, or one dilution, in preliminary studies.

### Structural modeling.

Structural homology models representing the capsid P domain of GII.4.2006A (GenBank accession number EF126964.1) and immunocompromised patient virus days 262 and 581 (31) were generated using Swiss-Model. To do this, capsid amino acid sequences for these viruses were uploaded into the Swiss-Model modeling server (https://swissmodel.expasy.org/interactive), and the appropriate background template was chosen by clicking “search for templates” and then choosing the known structure with the highest homology score. For all sequences, the chosen template was PDB accession number 3SLD, which is the crystal structure for the GII.4.2004 capsid bound to A trisaccharide (45). Models of the capsid dimers were created using the 3SLD template and downloaded in .pdb format. Models were rendered using MacPymol version 1.8.0.4 (https://www.pymol.org/). Hydrogen bonds were identified among specific amino acids by selecting those amino acids and using the “Action” command to choose “find,” then “polar contacts,” and then “within selection.” The distance in angstroms between epitopes E and F was calculated using Pymol’s measurement tool. The low end of the range was determined by measuring the distance between the closest residues within each epitope, and the high end of the range was the distance between the furthest residues within each epitope. To generate the full VLP image, the cryo-electron microscopy (cryo-EM) structure of GII.10 (EMD-5374) was downloaded from the Protein Data Bank in Europe (https://www.ebi.ac.uk/pdbe) and visualized in UCSF Chimera (version 1.12). The contour level was set R 2.87 and colored using the “surface color” function.
