# Supplementary figures and images for: Conformational Occlusion of Blockade Antibody Epitopes, a Novel Mechanism of GII.4 Human Norovirus Immune Evasion
Source: mSphere. 2018 Feb 7;3(1):e00518-17. doi: 10.1128/mSphere.00518-17 (PMC5806210; doi:10.1128/mSphere.00518-17)

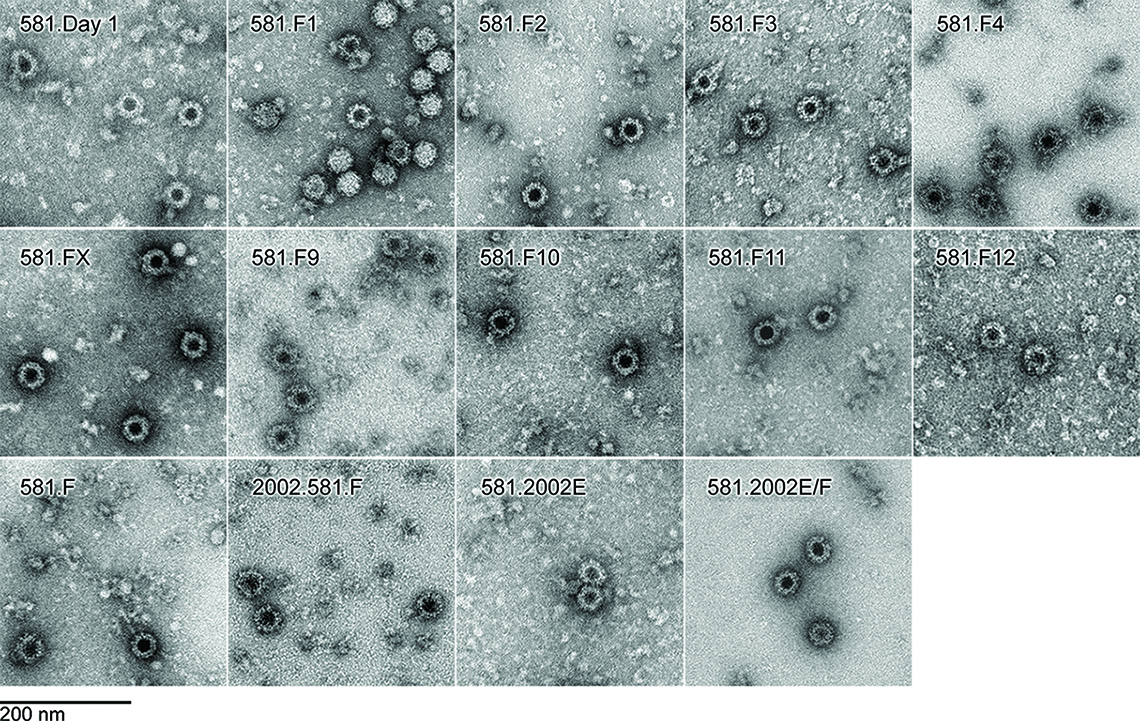

Supplement: FIG S1 [file sph001182475sf1.tif]
